# Supplementary material for: Are carbon emissions trading and green financial instruments synergistic? -Comprehensive quantitative research based on content analysis
Source: PLoS One. 2024 Mar 7;19(3):e0298601. doi: 10.1371/journal.pone.0298601 (PMC10919601; doi:10.1371/journal.pone.0298601)
Supplement: S1 Data — (ZIP) [file pone.0298601.s001.zip › Data/地级市Z_F工作报告.zip!/╒π╜¡/╠¿╓▌╩╨/└┤╫╘╣1⁄2╓┌║┼í╛┬φ┐╦╩2╛▌═°í┐_2016.docx]

2015年工作回顾和“十二五”发展成就

2015年，市政府认真落实市四届四次党代会决策部署，牢牢把握稳中求进总基调，主动适应经济发展新常态，积极融入国家战略新布局，围绕打造“一都三城”新目标，着力实施十大行动，统筹推进各项工作，实现了经济稳中有进、民生福祉增进、社会和谐稳定。

主要做了以下几方面工作：

（一）全力以赴稳增长。加强经济运行监测和统 计分析，出台工业、外贸、房地产等系列稳增长政策，深入开展“五帮一化”活动，联系帮扶企业“四个全覆盖”。实施重点项目挂图作战，推进“打霸拔钉、清障 护航”行动。加强要素保障。加大重大基础设施、重大产业、生态环保等项目投入。生产总值增长6.5%，固定资产投资增长13%，社会消费品零售总额增长 11%。

（二）千方百计调结构。首创科技大使制度。设立转型升级产业基金、产业并购基金、创业投资引导基金。入选国家产业振兴和 技术改造项目数、国家质量提升示范项目数、工信部首批智能制造项目数、全省高端装备制造业特色基地数、“浙江制造”认证企业数均居全省首位。新增上市企业 4家、新三板挂牌企业30家。启动“小微企业三年成长计划”，建设50个小微企业创业园。建筑业增加值增长9.3%。旅游人数、旅游总收入分别增长 22.0%和28.4%，天台山、神仙居景区同时跻身国家5A级景区行列。浙江中非国际经贸港落户。获批省级跨境电子商务试点市。网络零售额增长 45.7%。启动特色小镇培育创建“621”工程。市粮食储备配送中心基本建成。

（三）持之以恒抓改革。金融服务信用信息共享 平台建设深入推进，小微企业信保基金积极发挥作用，海峡两岸小微金融发展论坛成功举办，小微企业金融服务改革创新试验区成为一项国家战略举措。积极打造 “五张清单一张网”，在全省率先实行“一照一码”、“五证合一”和行政事业单位收费清单管理。

（四）坚定不移优环境。深入开展“三改一 拆”。强势推进劣Ⅴ类水质断面三年削减计划。县级以上饮用水源水质全部达标。成功创建省级节水型城市。交通治堵深入开展。全市空气质量优良天数比例达 90.1%。成为国家卫生城市、全国文明城市提名城市，国家环保模范城市、省级生态市通过验收。

（五）尽心尽力惠民生。完成十方面民生实事。 实现新农合与城镇居民基本医疗保险、被征地农民基本生活保障与职工养老保险、“老农保”与城乡居民养老保险并轨，开展机关事业单位人员养老保险缴存工作， 建立“基本医疗+大病保险”保障模式。新增城乡社区居家养老照料服务中心779家，居全省前列。新创建108所省级义务教育标准化学校。“双下沉、两提 升”工程全面实施，分级诊疗、全科医生签约服务稳步开展。成为国家公共文化服务体系示范区创建城市。农村应急广播建设实现全覆盖。成功举办首届台州国际马 拉松邀请赛。移民创业致富奔小康做法全国推广。低收入农户人均现金收入增幅达21.6%，居全省第1位。

（六）从严从实强法治。严格执行中央八项规定 精神，巩固党的群众路线教育实践活动成果，开展“三严三实”专题教育活动。自觉接受人大、政协和社会监督。出台重大行政决策工作规定，注重发挥决策咨询作 用。坚持每季度政府常务会议学法，出台行政应诉办法。深入开展“百日维稳攻坚大会战”等专项行动。“六五”普法和公共法律服务体系建设有序推进。开展千件 信访事项督查专项行动。12345政府热线积极受理解决群众诉求。加强食品药品安全、网络安全等问题治理。

刚刚过去的一年实现了“十二五”发展的顺利收官。

过去五年是经济运行较为稳健、综合实力不断提 升的五年。经济增长速度保持稳走向好的总体态势。2015年，生产总值达到3558.13亿元，年均增长7.5%；财政总收入和地方财政收入分别达到 539.78亿元、298.02亿元，年均增长11.7%和12.6%；固定资产投资五年累计7520.2亿元，年均增长20.2%；社会消费品零售总额 1826.68亿元，年均增长13.7%；进出口总额211.66亿美元，年均增长4.5%；城镇和农村常住居民人均可支配收入分别达到43266元、 21225元，年均增长10.1%和11.6%。

过去五年是产业结构调整力度加大、转型升级成 效明显的五年。三次产业比重调整到6.5：44.2：49.3。获得发明专利4226件，多次获得“全国科技进步先 进市”称号。36家上市公司成为引领转型的龙头，六大产业集群入选省级产业集群示范区试点，成为全国质量强市示范城市。建筑业产值突破2000亿元，成为 建筑强市。省市服务业集聚区加快建设，成功创建国家电子商务示范城市。农业“两区”建设成效明显。

过去五年是一批重大项目取得突破、基础设施持 续改善的五年。“十纵十横”公路网基本形成。沿海大通道全面贯通，沿海高速公路建设全线展开，金台铁路破土动工，台金高速东延二期开工建设，杭绍台铁路列 入国家社会资本投资铁路示范项目，市域铁路通过工可评审，朱溪水库获省可研批复。

过去五年是中心城市建设加快、城乡统筹协调发展的五年。城市化水平从55.5%提高到60.3%。中心城区建成区面积从116平方公里扩大到140平方公里。副中心城市、县域中心城市、小城市和中心镇加快培育。“百千”工程成效明显。

过去五年是环境质量大幅提升、生态文明扎实推 进的五年。“三改一拆”总量居全省前列。获省首批“五水共治”“大禹鼎”。主城区基本告别化工恶臭。成功创建国家循环经济示范城市。成为全国绿化模范城 市。长潭水库连续55个月保持Ⅰ类水质。省级生态市、省级森林城市实现全覆盖。节能减排任务超额完成。

过去五年是改革谋划最多、开放合作不断扩大的 五年。七大领域200项改革全面推进，获批国家部委改革试点34项，省级改革试点62项。行政审批“1+7”系列改革全省领先。商事登记改革全国领先。财 政专项资金竞争性分配和财政资金竞争性存放、公共资源配置差别化改革不断深化。台州湾产业集聚区东部新区启动区块基本成型。进出口企业诚信体系建设经验全 国推广。“浙商回归”工程到位资金909.02亿元。对口帮扶和对口支援工作扎实有效。

过去五年是民生持续改善、市民获得感明显增强 的五年。财政对民生投入1235亿元，年均增长18.3%。新增就业人数40万人，新增基本养老、基本医疗保险参保人数140万人和115.2万人，新增 住房公积金缴存人数26.8万人。发放慈善资金6.8亿元，帮助困难群众45.9万人。全面消除家庭人均收入4600元以下贫困户，欠发达县完成“摘 帽”。义务教育完成率99.9%，高中段教育毛入学率97.5%，高等教育毛入学率53%。医疗资源“三千提增计划”深入实施。国家公共文化服务体系示范 区创建扎实推进。食品安全基层责任网和放心市场、快检室、阳光厨房建设全省领先。连续4次获得全国双拥模范城称号。

“十三五”奋斗目标和战略重点

“十三五”工作的指导思想：高举中国特色社会 主义伟大旗帜，以马列主义、毛泽东思想、邓小平理论、“三个代表”重要思想、科学发展观为指导，深入贯彻习近平总书记系列重要讲话精神，以“四个全面”战 略布局为统领，以创新、协调、绿色、开放、共享五大发展理念为引领，以“八八战略”为总纲，以“干在实处永无止境，走在前列要谋新篇”为新使命，以“更进 一步、更快一步，继续发挥先行和示范作用”为总要求，紧紧围绕“一都三城”战略目标，着力推动“三区”融合、“三市”同城、“三湾”联动、“三带”协同， 坚持发展是第一要务，提高发展质量和效益，加快形成引领经济发展新常态的体制机制和发展方式，统筹推进经济、政治、文化、社会、生态文明建设和党的建设， 确保实现“四翻番”，高水平全面建成小康社会。

“十三五”发展的主要目标。总体目标是拉高标 杆、争先进位，确保实现“四翻番”，高质量构建经济发展新体系，着力推进“民营经济创新发展、综合交通等基础设施建设、补齐关键领域短板”三大历史任务， 高水平全面建成小康社会。具体目标：一是综合实力更强大，战略地位进一步提升。二是城乡区域更协调，都市区发展格局基本形成。三是创新动能更强劲，国际智 造名城建设取得明显成效。四是开放平台更广阔，海上丝路港城建设取得重大进展。五是人民生活更幸福，山海宜居美城建设取得崭新成果。六是治理体系更完善， 全面深化改革取得重大突破。

开拓发展新境界，实现“十三五”目标，必须集中力量抓好既该干又能干成的大事，争做全省发展民营经济的排头兵，早日跻身全省经济总量的第二方阵，努力当好我省打造全面小康标杆省份的中坚力量。

（一）着力推进三大历史任务。一是加快打造先进制造业基地，推进台州民营经济再创新辉煌。二是加快以综合交通为重点的基础设施建设，实现升级版的互联互通。三是加快补齐关键领域短板，提高发展的协调性和平衡性。

（二）全面推进都市区建设。一是强化中心城市，推进“三区”融合。二是联动副中心城市，推进“三市”同城。三是统筹“四县”，推进市县协同。四是建设小城市，推进中心镇发展。五是建设美丽宜居乡村。

（三）建设湾区经济发展试验区。一是按照“山 海统筹、临港布局、拥湾发展、串珠成链”的整体思路，加大汽车、电力能源储运、大宗商品交易平台等临港大项目大产业的引进力度，构建以创新、开放、宜居、 国际化为特征的现代湾区经济体系。二是加快建立“三湾”联动的湾区经济发展机制，重点谋划港口联动、道路联动、产业联动、机制联动，努力将“三湾”打造成 产业集聚区、科技引领区、众创先行区、生态示范区。三是启动湾区发展规划，保护湾区岸线生态资源。四是以开放包容的姿态深化与周边的合作开发。

（四）完善现代产业体系。一是重点培育现代医 药、汽车制造、高端装备、清洁能源、信息经济、现代金融、现代物流、旅游休闲等八大千亿主导产业。二是重点培育航空产业、新材料、节能环保、海洋新兴、时 尚产业、文化创意、健康养生、现代商务等八大百亿新兴产业。三是重点推进缝制设备、休闲用品、塑料模具、家用电器、服装鞋帽、再生资源、建筑业、船舶修造 等八大传统产业转型升级。振兴“一线四雕”等历史经典产业。加快大数据产业发展和云平台建设，推动“互联网+”和“+互联网”行动，以“互联网+”带动新 兴产业发展。

（五）打造六大发展平台。一是科技创新平台。二是产业发展平台。三是城市建设平台。四是港区开放平台。五是特色小镇平台。六是绿色生态平台。

（六）实施“万亿投资”计划。规划建设重大项目982个，总投资1.76万亿元，五年计划投资超9000亿元，总投资100亿元以上的项目32个。

（七）全面深化“5+4”重点改革。一是以中 央全面深化改革决定为统领，深入推进政府、经济、城乡、生态、社会五大重点领域和关键环节改革，着力突破体制机制障碍。二是以国家赋予台州改革试点为契 机，加快小微企业金融服务改革创新试验区、新型城镇化综合试点、民间投资创新综合改革试点、循环经济示范城市建设等四大国家级改革试点。

（八）优化五大发展环境。一是优化人才人文环境。二是优化金融等要素环境。三是优化政务生态环境。四是优化信用法治环境。五是优化绿色生态环境。

2016年主要任务

今年是“十三五”开局之年。建议经济社会发展 的主要目标为：地区生产总值增长7%以上，固定资产投资增长11%，社会消费品零售总额增长10%，货物出口总额保持适度增长，地方财政收入增长7%，城 乡常住居民人均可支配收入分别增长8%和9%，单位生产总值能耗下降和主要污染物减排完成省定目标。

为实现今年目标，重点抓好以下六项任务：

（一）强化创新驱动，推进经济转型升级

加快科技创新。发挥企业创新主体作用，加大创 新投入，研发经费支出67亿元。完成台州科技城规划设计，启动孵化园及公共服务区建设。建立科技指导员制度。新认定高新技术企业20家以上，培育科技型中 小企业200家。创建省级创新型城市和国家知识产权示范城市。启动人才管理改革试验区建设，完善“人才项目+民企民资”创业合作模式。

推动产业升级。制定“中国制造2025”台州 行动计划。深化“四换三名”工程。推进“小微企业三年成长计划”。深化质量强市示范城市建设。积极引进和建设中车配套产业园、航天科技无人机生产基地、金 属燃料电池加工基地和中德小微企业合作基地。加快新型建筑工业化发展。大力发展现代服务业，推动服务业强县（市、区）培育。进一步发展金融业。加快电子商 务发展，争取列入国家跨境电商试点城市。着力推动全域景区化，谋划打造沿海连岛旅游精品线。大力发展现代物流业和会展经济。培育健康养老、时尚经济、科技 服务等新兴产业。

（二）提高供给体系质量和效率，保持经济平稳运行

积极扩大有效投资。主攻项目，重点实施“十大 标志工程”，即杭绍台铁路、金台铁路、市域铁路、沿海高速、杭绍台高速、台州新机场、商贸核心区、台州科技城、台州大剧院、栅岭汪和洪家场浦排涝调蓄工 程。建立多元化投融资机制，加快推广PPP模式，推进产业基金实质性运作。积极破解耕地占补平衡难题。

着力推进“三去一降”和扩大内需。积极稳妥化解过剩产能。化解房地产库存，调控土地供给量。推进住房征收市场化安置，构建多层次住房保障体系。建立经济风险预警机制，主动释放信用违约风险信号。

大力拓展国际市场。扩大与“一带一路”沿线国 家的经贸合作。支持企业参加60个境内外重点展会。鼓励有实力的企业从商品出口向“商品+服务”出口转变，积极发展运输、建筑等服务贸易。支持企业设立公 共海外仓、体验店。争取列入国家市场采购政策试点。加快浙江中非国际经贸港建设，用足用好出口信用保险政策，助推中小企业加大出口。大力推动国际并购，参 与国际产能合作。优化进口结构，加强先进技术、关键设备、能源资源进口。提升进出口企业诚信建设水平。

（三）不断深化各项改革，扩大开放合作

扎实推进各项改革。制定实施小微企业金融服务 改革创新试验区建设方案。推进国家民间投资创新综合改革试点、国家循环经济示范城市、国家新型城镇化综合试点城市。深化“五张清单一张网”建设。深化商标 权质押登记改革。稳妥实施环保机构执法垂直管理制度。加强行政审批事中事后监管。进一步清理规范中介机构。加快建立统一的公共资源交易平台。推进国资国企 改革。推进市区一体化改革。加快综合执法体制、不动产统一登记和县域经济体制综合改革。稳妥推进户籍制度改革。全面开展农村土地承包经营权确权赋权登记颁 证工作。

大力推动开放合作。推进湾区经济试验区建设。 台州经济开发区创建国家级高新技术产业园区。加快头门港区口岸开放步伐。推进国际贸易单一窗口和椒江、临海、温岭、玉环、天台公用型保税仓库建设。加快对 台经贸合作区、海峡两岸（玉环）商品交易物流中心建设。加强与世界500强、央企、军企的战略合作，深入实施“浙商回归”工程。

（四）统筹城乡发展，建设美丽台州

推进都市区建设。树立城市“精明增长”理念， 全面开展总规修改，实施“多规合一”。集聚区加快“一湖三园一基地”建设。加快建设内环快速路、开发大道东延、聚洋大道等，开工建设现代大道，建成台州大 道。争取台州火车站获批始发站。完善市区椒江两岸规划。开展海绵城市建设试点。推进智慧城市建设。加强市容市貌管理。推进特色小镇建设。

加强农业农村工作。大力推进农业现代化。加快 全产业链建设和农产品质量建设、品牌建设，加快现代农业标准化示范项目建设，推进农作制度示范乡镇创建。实施农业“两区”绿色发展三年行动计划。大力发展 现代种业。扶持发展农业龙头企业、家庭农场（股份合作农场）。大力发展生态渔业和远洋渔业，推进渔场修复振兴。提高新农村建设水平。深入推进美丽乡村精品 村、历史文化村落创建。积极发展休闲农业和民宿经济，推进乡村旅游休闲业提档升级。加快培养新型职业农民。

推进生态环境建设。制定实施新一轮“811” 美丽台州建设行动。实施主体功能区规划和环境功能区划，重点环境功能区实行产业准入负面清单。加快节能减排重点项目建设。大力推进国家森林城市创建。加快 仙居国家公园、省级绿色化改革试点。系统推进专项组合拳。深入开展“五水共治”，大力推进劣Ⅴ类水质断面三年削减计划。巩固重点行业整治提升成果，深化大 气污染防治专项行动。开展土壤污染治理与修复，确保耕地利用安全。实施蓝色海湾整治行动，打造滨海生态走廊。完善“三改一拆”长效工作机制，深入开展无违 建县（市、区）创建。推进交通治堵。

（五）加大民生补短力度，保障社会安定有序

大力发展社会事业。全面推进教育现代化县 （市、区）创建，实现“基础教育重点县”摘帽。深化医药卫生体制改革。稳妥实施“全面两孩”政策。深入开展全国文明城市创建工作。推进城市文化公园、企业 文化俱乐部、农村文化礼堂、社区文化家园建设，推进媒体融合发展。开展全民健身运动。进一步夯实统计基础，认真做好第三次农业普查工作。

完善社会保障体系。深入实施就业优先战略。实 施医保阳光智能监管。促进市区城乡低保标准一体化。完善最低工资保障机制。加快养老综合体建设。保障妇女儿童权益。建设社会福利机构。发扬慈孝文化，发展 慈善事业。高度关注最困难群体、因灾因病返贫群体和困难家庭下一代教育，深入实施“低收入农户收入倍增计划”，全面推进移民创业致富奔小康行动。

创新社会治理体制。推进“平安台州”建设。推 进信访源头管控和程序改革。启动“七五”普法工作。深入开展移风易俗活动。加强基层政权建设。实施政务服务热线整合。建成市政府应急指挥中心。推进食品药 品、特种设备“智慧监管”系统建设。建立健全征信体系。强化安全生产主体责任和监管责任。推进军民融合深度发展。

（六）加强政府自身建设，提高政府治理现代化水平

创新政府管理方式。扩大政府购买公共服务的领域和范围。推进各领域决策、执行、管理、服务、结果等环节政务公开。落实政府新闻发言人和突发事件信息发布制度。引入第三方评估，促进政府管理创新。

强化行政权力制约和监督。依法接受人大及其常委会的法律监督和工作监督，自觉接受人民政协的民主监督。健全依法决策机制。强化行政监察，加大审计监督力度。健全重大决策失误责任倒查机制和追责制度。

推进政风改进常态化。认真履行“一岗双责”， 认真执行《中国共产党廉洁自律准则》《中国共产党纪律处分条例》和市委相关要求。坚持用制度管权管事管人，坚持零容忍惩治腐败。强化“实绩论英雄”导向， 完善政绩考核评价机制。巩固党的群众路线教育实践活动和“三严三实”专题教育成果，健全作风建设长效机制。加强政府效能建设，加大问效问责力度。
